# Lorentzian-Corrected Apparent Exchange-Dependent Relaxation (LAREX) Ω-Plot Analysis—An Adaptation for qCEST in a Multi-Pool System: Comprehensive In Silico, In Situ, and In Vivo Studies

**DOI:** 10.3390/ijms23136920

**Published:** 2022-06-22

**Authors:** Karl Ludger Radke, Lena Marie Wilms, Miriam Frenken, Julia Stabinska, Marek Knet, Benedikt Kamp, Thomas Andreas Thiel, Timm Joachim Filler, Sven Nebelung, Gerald Antoch, Daniel Benjamin Abrar, Hans-Jörg Wittsack, Anja Müller-Lutz

**Affiliations:** 1 Department of Diagnostic and Interventional Radiology, Medical Faculty, University Dusseldorf, D-40225 Dusseldorf, Germany; ludger.radke@med.uni-duesseldorf.de (K.L.R.); miriam.frenken@med.uni-duesseldorf.de (M.F.); marek.knet@hhu.de (M.K.); benedikt.kamp@med.uni-duesseldorf.de (B.K.); thomas.thiel@med.uni-duesseldorf.de (T.A.T.); snebelung@ukaachen.de (S.N.); antoch@med.uni-duesseldorf.de (G.A.); danielbenjamin.abrar@med.uni-duesseldorf.de (D.B.A.); hans-joerg.wittsack@med.uni-duesseldorf.de (H.-J.W.); anja.lutz@med.uni-duesseldorf.de (A.M.-L.); 2 F.M. Kirby Center for Functional Brain Imaging, Kennedy Krieger Institute, Baltimore, MD 21205, USA; stabinska@kennedykrieger.org; 3 Department of Radiology, Johns Hopkins University School of Medicine, Baltimore, MD 21205, USA; 4 Institute of Anatomy I, Heinrich-Heine-University, D-40225 Dusseldorf, Germany; timm.filler@uni-duesseldorf.de; 5 Department of Diagnostic and Interventional Radiology, University Hospital Aachen, D-52074 Aachen, Germany

**Keywords:** magnetic resonance imaging, molecular imaging, CEST, qCEST, in situ, in vivo, in silico, Bloch–McConnell, IVD

## Abstract

Based on in silico, in situ, and in vivo studies, this study aims to develop a new method for the quantitative chemical exchange saturation transfer (qCEST) technique considering multi-pool systems. To this end, we extended the state-of-the-art apparent exchange-dependent relaxation (AREX) method with a Lorentzian correction (LAREX). We then validated this new method with in situ and in vivo experiments on human intervertebral discs (IVDs) using the Kendall-Tau correlation coefficient. In the in silico experiments, we observed significant deviations of the AREX method as a function of the underlying exchange rate (k_ba_) and fractional concentration (f_b_) compared to the ground truth due to the influence of other exchange pools. In comparison to AREX, the LAREX-based Ω-plot approach yielded a substantial improvement. In the subsequent in situ and in vivo experiments on human IVDs, no correlation to the histological reference standard or Pfirrmann classification could be found for the f_b_ (in situ: τ = −0.17 *p* = 0.51; in vivo: τ = 0.13 *p* = 0.30) and k_ba_ (in situ: τ = 0.042 *p* = 0.87; in vivo: τ = −0.26 *p* = 0.04) of Glycosaminoglycan (GAG) with AREX. In contrast, the influence of interfering pools could be corrected by LAREX, and a moderate to strong correlation was observed for the fractional concentration of GAG for both in situ (τ = −0.71 *p* = 0.005) and in vivo (τ = −0.49 *p* < 0.001) experiments. The study presented here is the first to introduce a new qCEST method that enables qCEST imaging in systems with multiple proton pools.

## 1. Introduction

Low back pain is one of the most common health concerns worldwide, with a lifetime prevalence of up to 80% and a huge impact on patients’ quality of life and socioeconomic status [1]. Degenerative disc disease (DDD) is believed to be the main cause of low back pain [2]. However, the underlying degenerative processes of DDD are not yet fully understood. While clinical-standard magnetic resonance imaging (MRI) is the most sensitive imaging method to assess DDD by depicting structural damage, early alterations cannot be quantified [3,4]. Consequently, more sensitive biochemical techniques are needed to assess the underlying biochemical changes that develop during the early stages of cartilage degeneration.

Chemical exchange saturation transfer (CEST) imaging has emerged as a promising bio-sensitive MRI technique alongside Na-, T1ρ-, and diffusion tensor imaging [3,5,6,7]. In musculoskeletal imaging, CEST has been used to assess the biochemical composition of the intervertebral discs (IVDs) [3,4,8]. The early detection of degenerative IVD alterations using CEST imaging may allow for the timely diagnosis of degenerative diseases, the initiation of targeted therapy, and a better understanding of degenerative processes. Glycosaminoglycans are an important component of the intervertebral discs and form the side chains of the complex proteoglycan molecule [9]. They are linear polysaccharides, which are composed of disaccharide units. Each of these disaccharide units has three hydroxyl and one NH group [10]. The intervertebral discs themselves consist of the collagen-fibrous annulus fibrosus and the nucleus pulposus, which is predominantly composed of proteoglycans [11]. They serve to soften shocks as well as to distribute pressure to adjacent vertebral bodies. Glycosaminoglycans are essential for this function [11].

By assessing changes in the water volume signal after selective saturation, CEST imaging is sensitive to the detection of small amounts of labile protons [12,13], and thus provides essential information that can complement conventional morphologic MRI methods. Particularly, the CEST contrast is approximately proportional to the concentration and exchange rate of the observed labile protons [14]. In fact, the measured CEST effects vary with the fractional concentration of labile protons and the exchange rate and depend on other parameters such as radiofrequency (RF) power, B_0_ field strength, regional pH value, temperature, and T_1_ and T_2_ relaxation times [15,16,17].

CEST imaging techniques have further evolved over the last few years due to contributions from various technical fields, including the development of mathematical models and hardware improvement [18,19]. Furthermore, methods for the simultaneous determination of fractional concentrations and exchange rate have been introduced [17,20,21]. Based on these new methods, CEST imaging has developed into quantitative CEST analysis (qCEST) [18]. Among others, Dixon et al. indicated that the CEST effect can be plotted as a linear function of 1/B_1_ (Ω-plot) and that, consequently, the proton exchange rate (k_ba_) from Pool B to Pool A and the labile proton ratio (f_b_) of Pool B can be determined by linear regression [20]. However, this method is limited to paramagnetic CEST agents, which can be considered independent of direct water saturation (so-called spillover effect) and the magnetization transfer (MT) effect due to the large chemical shift to water [22]. For the qCEST imaging of endogenous CEST agents with smaller chemical shifts (<5 ppm), Meissner et al. proposed a novel quantitative CEST-MRI method termed AREX (apparent exchange dependent relaxation)-based Ω-plots [21]. This metric uses the MTR_REX_ calculation introduced by Zaiss et al., eliminates the spillover and MT effects, and extends it with a T_1_ relaxation component [23]. However, while this approach is based on the assumption of a two-pool system, most human biochemical systems interact with more than one labile proton pool. Consequently, many studies have extended the conventional asymmetry analysis (MTR_asym_) to a multi-Lorentzian analysis [19,24,25,26], allowing the separate determination of different pools’ effects. Assuming that each saturation transfer and semi-solid MT signal can be approximated as a Lorentzian lineshape, these various pool effects are considered and corrected [27]. Recently, the first studies have demonstrated an increased accuracy in detecting both the MTR_asym_ and AREX signals based on multi-pool Lorentzian fits in simulations and animal tumor models [25]. Yet, to our knowledge, no prior study has used the separation of individual exchange processes via Lorentzian analysis of different B_1_ values to extend AREX-based Ω-plot analysis.

Therefore, the present study aimed to (a) develop a CEST quantification algorithm applicable to multi-pool systems called Lorentzian-corrected apparent exchange-dependent relaxation (LAREX)-based Ω-plot analysis, and (b) validate the presented algorithm by in situ and in vivo experiments for several medically relevant multi-pool systems, such as amide proton transfer (APT, frequency offset Δs = 3.5 ppm) determination of the white matter in the visual cortex and histological determination of glycosaminoglycan (GAG, Δs = 1 ppm) concentration in human IVDs. Additionally, in vivo experiments are used to illustrate the transferability of the in situ results to real MR measurements. To this end, we hypothesized that (a) the AREX-based Ω-Plot method cannot be readily transferred from a two-pool system to a multi-pool system, (b) the introduced LAREX method can be used to separate the different pools and achieve comparable accuracy concerning the parameters f_b_ and k_ba_ as AREX in an ideal two-pool system, and (c) that conventional AREX-based evaluation cannot detect degeneration both in situ and in vivo due to the precision of different pools in human IVDs, but that these effects can be separated and subsequently evaluated using our LAREX approach.

## 2. Results

### 2.1. In Silico Study

**Figure 1:**
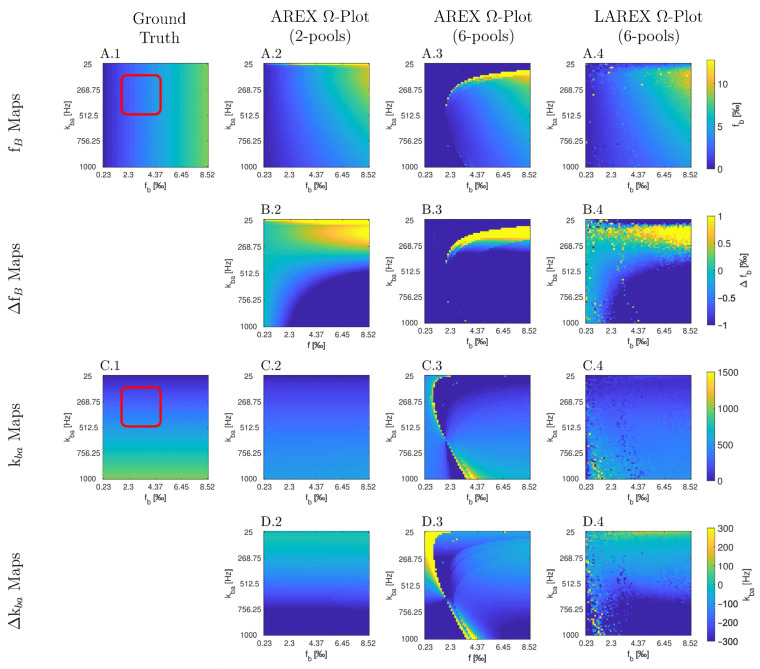
Parameter maps (**A**,**C**) and color-coded error maps (**B**,**D**) for fractional Glycosaminoglycan (GAG) concentrations f_b_ and exchange rates k_ba_, as a function of f_b_ and k_ba_, determined by apparent exchange-dependent relaxation (AREX) Ω-plots on two- and six-pool systems, LAREX Ω-plots on a six pool system, as well as the ground truth maps with a red box with marked the clinically relevant physiological range of IVD changes. The physiological parameters used are based on the exchange rates and relaxation times of human IVDs at 3 Tesla observed in previous studies [8,25,28], and are listed separately in Table 1. In this case, we only used the LAREX-based evaluation for the six-pool system because the separation of different pools characterizes this approach. The calculations are then based on the two-pool system separated in this way. Corresponding figures for further multi-pool systems investigated, such as APT qCEST imaging in the human brain, are provided in Appendix A.

**Figure A1:**
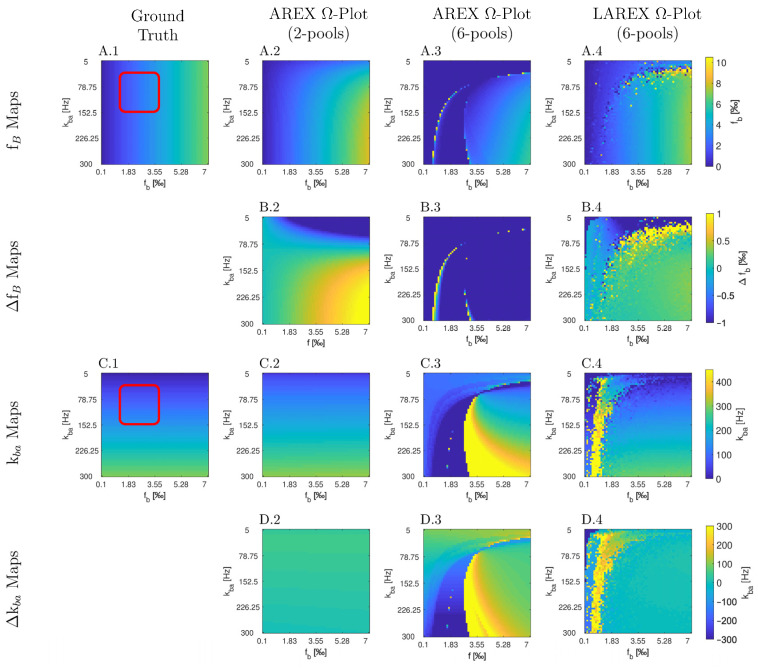
In silico Appendix Study 1—APT qCEST analyses of white matter of the visual cortex. Parameter maps (**A**,**C**) and color-coded error maps (**B**,**D**) for fractional APT concentrations f_b_ and exchange rates k_ba_ as a function of f_b_ and k_ba_. The study was performed using a 4-pool system and a field strength of 7 Tesla. The parameters of the exchanging proton pools were: Water (T_1_ = 1200 ms, T_2_ = 40 ms, and Δ = 0 ppm); APT (T_1_ = 1000 ms, T_2_ = 10 ms, f = variable between 0.001–0.2, k_ba_ = variable between 1–300 Hz, and Δ = 3.5); NOE (T_1_ = 1000 ms, T_2_ = 0.3 ms, f_s_ = 0.06, k_ba_ = 10 Hz, and Δ_s_ = −3.5 ppm); MT (T_1_ = 1000 ms, T_2_ = 10 µs, f_s_ = 0.11, k_ba_ = 50 Hz, and Δ_s_ = 0 ppm), and were based on the results of Mougin et al. [67]. Furthermore, analogous to the in silico, in situ, and in vivo experiments, the same MR parameters (TR = 2500 ms, TE = 3.5 ms) and presaturation parameters (n_p_ = 40, t_p_ = 100 ms, t_d_ = 100 ms, presaturation pulse shape = Gaussian, Δω = 5 ppm) were used.

**Figure A2:**
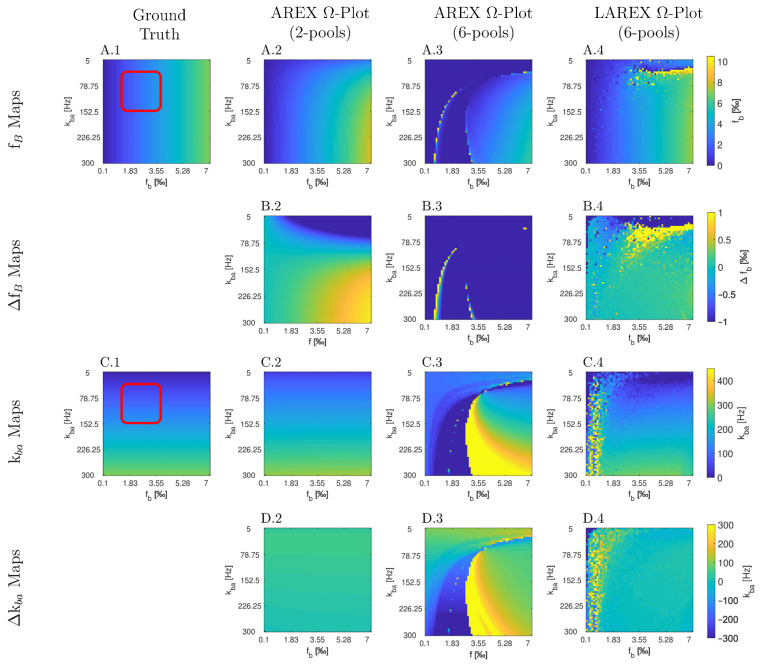
In silico Appendix Study 2—APT qCEST analyses of gray matter of the visual cortex. Parameter maps (**A**,**C**) and color-coded error maps (**B**,**D**) for fractional APT concentrations f_b_ and exchange rates k_ba_ as a function of f_b_ and k_ba_. The study was performed using a 4-pool system and a field strength of 7 Tesla. The parameters of the exchanging proton pools were: Water (T_1_ = 2000 ms, T_2_ = 55 ms, and Δ = 0 ppm); APT (T_1_ = 1000 ms, T_2_ = 10 ms, f = variable between 0.001–0.01, k_ba_ = variable between 0–300 Hz, and Δ = 3.5); NOE (T_1_ = 1000 ms, T_2_ = 0.3 ms, f = 0.06, k_ba_ = 10 Hz, and Δ_s_ = −3.5 ppm); MT (T_1_ = 1000 ms, T_2_ = 10 µs, f = 0.11, k_ba_ = 50 Hz, and Δ = 0 ppm), and were based on the results of Mougin et al. [67]. Furthermore, analogous to the in silico, in situ, and in vivo experiments, the same MR parameters (TR = 2500 ms, TE = 3.5 ms) and presaturation parameters (n_p_ = 40, t_p_ = 100 ms, t_d_ = 100 ms, presaturation pulse shape = Gaussian, Δω = 5 ppm) were used.

**Figure A3:**
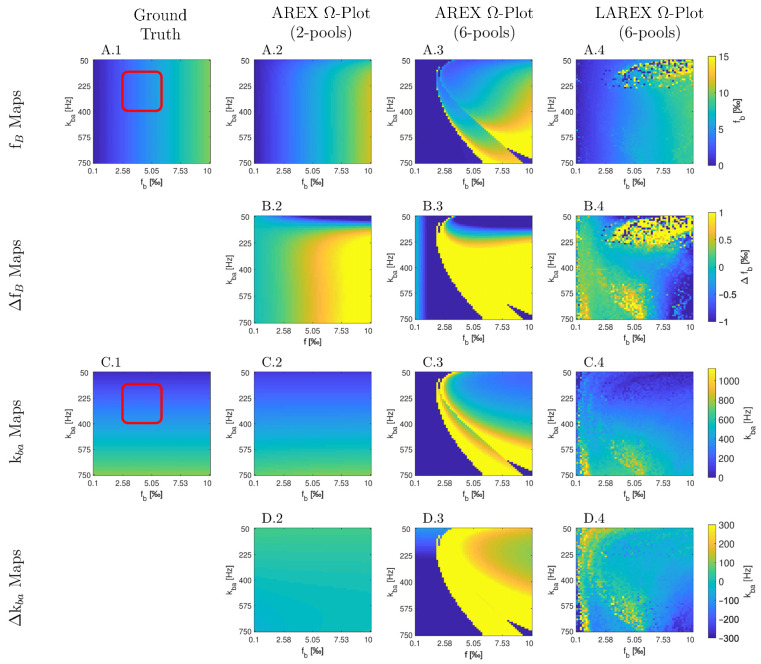
In silico Appendix Study 3—Amine qCEST analyses of the human ex-vivo blood. Parameter maps (**A**,**C**) and color-coded error maps (**B**,**D**) for fractional Amine concentrations f_b_ and APT exchange rates k_ba_ as a function of f_b_ and k_ba_. The study was performed using a 6-pool system and a field strength of 7 Tesla. The parameters of the exchanging proton pools were: Water (T_1_ = 2249 ms, T_2_ = 48.5 ms, and Δ_s_ = 0 ppm); Amine (T_1_ = 1000 ms, T_2_ = 10 ms, f = variable 0.0001–0.01, k_ba_ = variable 50–750 Hz and Δ = 2.2); APT (T_1_ = 1000 ms, T_2_ = 0.5 ms, f = 0.0078, k_ba_ = 338.7 and Δ_s_ = 3.5); NOE #1 (T_1_ = 1000 ms, T_2_ = 1.2 ms, f = 0.018, k_ba_ = 10 Hz and Δ = −1.7 ppm); NOE #2 (T_1_ = 1000 ms, T_2_ = 0.5 ms, f = 0.0144, k_ba_ = 5 Hz and Δ_s_ = −3.5); MT (T_1_ = 1000 ms, T_2_ = 0.01 ms, f = 0.0105, k_ba_ = 15 and Δ = −2.34), and were based on the results of Shah et al. [68]. Furthermore, analogous to the in silico, in situ, and in vivo experiments, the same MR parameters (TR = 2500 ms, TE = 3.5 ms) and presaturation parameters (n_p_ = 40, t_p_ = 100 ms, t_d_ = 100 ms, presaturation pulse shape = Gaussian, Δω = 5 ppm) were used.

**Figure A4:**
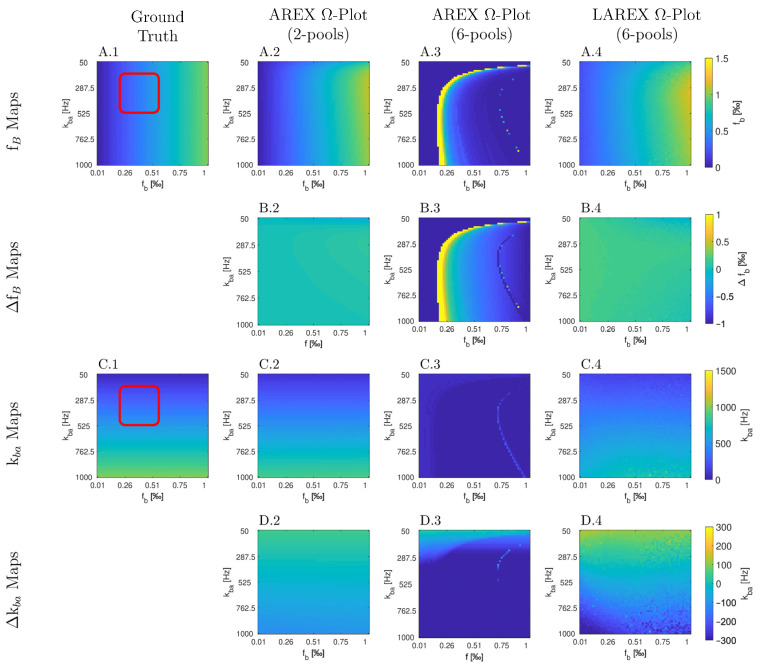
In silico Appendix Study 4—Creatine qCEST analyses of human brain. Parameter maps (**A**,**C**) and color-coded error maps (**B**,**D**) for fractional Creatine concentrations f_b_ and Creatine exchange rates k_ba_ as a function of f_b_ and k_ba_. The study was performed using a 6-pool system and a field strength of 7 Tesla. The parameters of the exchanging proton pools were: Water (T_1_ = 2000 ms, T_2_ = 115 ms, and Δ = 0 ppm); Creatine (T_1_ = 1000 ms, T_2_ = 10 ms, f = variable 0.00001–0.001, k_sw_ = variable 50–1000 Hz and Δ = 1.8); Amide (T_1_ = 1000 ms, T_2_ = 33 ms, f = 0.0008, k_ba_ = 30 and Δ = 3.5); Glutamate (T_1_ = 1000 ms, T_2_ = 10 ms, f_s_ = 0.000125, k_sw_ = 2000 and Δ_s_ = 3.0); Myo-inositol (T_1_ = 1000 ms, T_2_ = 10 ms, f = 0.0007, k_ba_ = 600 and Δ = 0.6); NOE (T_1_ = 1000 ms, T_2_ = 0.01 ms, f = 0.0795, k_ba_ = 10 and Δ = −2.4), and were based on the results of Sing et al. [69]. Furthermore, analogous to the in silico, in situ, and in vivo experiments, the same MR parameters (TR = 2500 ms, TE = 3.5 ms) and presaturation parameters (n_p_ = 40, t_p_ = 100 ms, t_d_ = 100 ms, presaturation pulse shape = Gaussian, Δω = 5 ppm) were used.

The Bloch–McConnell simulation (please refer to Table 1 for an overview of all the parameters used) over an extended physiological range of fractional concentrations (f_b_) and exchange rates (k_ba_) demonstrated substantial differences in accuracy and feasibility between AREX- and LAREX-based Ω-plots (Figure 1). To better illustrate the accuracy of the methods and to contextualize them in the AREX-based Ω-plot in the two-pool system, Δf_b_ (Figure 1A) and Δk_ba_ maps (Figure 1C) were calculated with a color-coded error range of Δf_b_ = ±1‰ (Figure 1B) and Δk_ba_ = ±300 Hz (Figure 1D). The AREX approach did not accurately display the full analyzed range of f_b_ and k_ba_. Our proposed LAREX approach allows for widely sufficient accuracy for both k_ba_ value and fractional concentration f_b_ determination. However, analogous to the AREX approach for two-proton pools, the exchange rate k_ba_ and the fractional concentration f_b_ tend to be underestimated for fast exchange rates. Similar findings were observed for our APT-qCEST models of the white and gray matter of the visual cortex (Appendix A Figure A1 and Figure A2), our amine-qCEST model of ex-vivo blood (Figure A3), and for our creatine qCEST brain model (Figure A4).

### 2.2. In Situ Study

**Figure 2:**
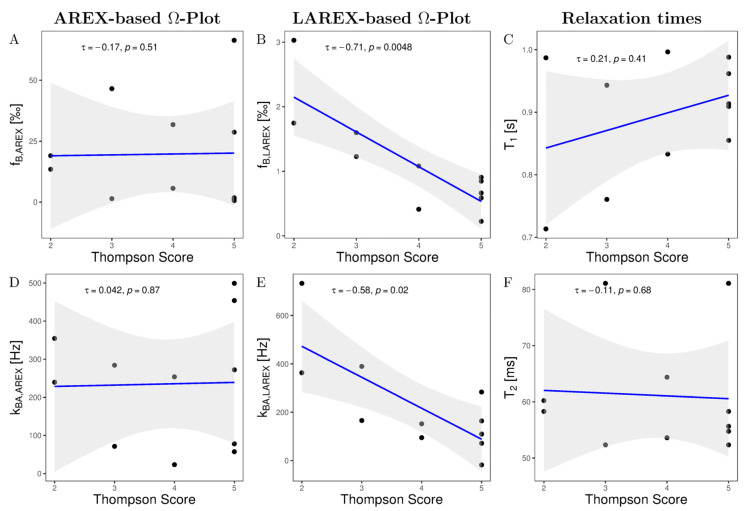
Kendall-Tau correlations of altered GAG fractional concentrations f_b_ (**A**,**B**), T_1_ relaxation times (**C**), GAG exchange rates k_ba_ (**D**,**E**), and T_2_ relaxation times (**F**) as a function of Thompson scoring in our measured in situ IVDs. In all correlation diagrams, a black dot corresponds to the mean value of the studied parameter for a single IVD and the gray background illustrates the 95% confidence interval of the correlation. (**A**) AREX-based fraction concentrations show a non-significant and negligible decrease with Thompson scoring. Moreover, AREX-based fractional concentrations scatter in a non-physiological range. (**B**) The LAREX-based determination shows a strong and significant decrease as a function of Thompson scoring. (**C**,**F**) Both Relaxation times show a non-significant correlation as a function of Thompson scoring. (**D**) AREX-based exchange rate determination shows no correlation as a function of Thompson rating. (**E**) The LAREX-based determination shows a strong and significant decrease as a function of the Thompson rating.

Ω-plots derived from eleven IVDs based on AREX and LAREX were calculated and histologically referenced. Based on the Thompson classification [29], degeneration of IVDs was quantified as follows: Grade 1 (n = 0), Grade 2 (n = 2), Grade 3 (n = 2), Grade 4 (n = 2), and Grade 5 (n = 5). In the AREX-based Ω-analysis, we did not find a significant correlation for neither fractional concentrations (τ = −0.17, *p* = 0.51; Figure 2A) nor exchange rates (τ = 0.042, *p* = 0.87; Figure 2B). It should be mentioned that the fractional concentration for AREX-based Ω-analysis scatter in a non-physiological range. In contrast, we observed a strong and significant decrease in f_b_ (τ = −0.71, *p* = 0.0048, Figure 2C) and a strong but non-significant decrease in k_ba_ (τ = −0.58, *p* ≤ 0.02, Figure 2D) with LAREX-based Ω assessment. Furthermore, the f_B_ values were with f_B_ 0–3.5 in a physiological-range. In comparison, we observed no significant correlations as a function of IVD degeneration for either T_1_ or T_2_ relaxation times.

For the AREX-based Ω-plot analyses, an excellent reliability was observed for the determination of fractional concentration with ICC(3,1) = 0.99 (95% CI = 0.98–1) and ICC(2,1) = 0.98 (95% CI = 0.93–0.99). In addition, the determination of the exchange rate showed excellent agreement, respectively, with ICC(3,1) = 0.98 (95% CI = 0.98–1) and ICC(2,1) = 0.98 (95% CI = 0.98–1). Similarly, for our proposed LAREX-based Ω-plot analysis, we found excellent and good reliability with ICC(3,1) = 0.91 (95% CI = 0.76–0.97) and ICC(2,1) = 0.97 (95% CI = 0.91–0.99) for fractional concentration and ICC(3,1) = 0.97 (95% CI = 0.91–0.99) and ICC(2,1) = 0.68 (95% CI = 0.37–0.85) for exchange rate observation.

### 2.3. In Vivo Study

**Figure 3:**
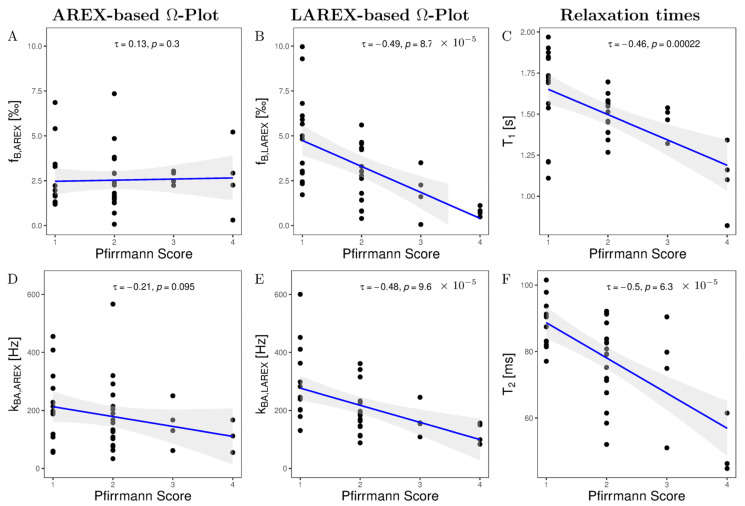
Kendall-Tau correlations of altered fractional GAG concentrations f_b_ (**A**,**B**), GAG exchange rates k_ba_ (**D**,**F**), T_1_- (**C**), and T_2_ relaxation times (**F**) as a function of Pfirrmann scoring in our measured in vivo IVDs and the two methods examined, the AREX-based Ω-plot and the LAREX-based Ω-plot. In all correlation plots, a black dot corresponds to the mean value of the studied parameter for a single IVD and the gray background illustrates the 95% confidence interval of the correlation. (**A**) AREX-based fractional concentrations show a non-significant and negligible decrease with Pfirrmann scoring. (**B**) The LAREX-based determination of fractional GAG concentrations shows a moderate and significant decrease as a function of Pfirrmann scoring. (**C**) T_1_ relaxation times shows a moderate and significant decrease with Pfirrmann scoring. (**D**) The AREX-based exchange rate determination shows a small and non-significant increase as a function of Pfirrmann scoring. (**E**) With Pfirrmann scoring, the LAREX-based exchange rate shows a moderate and significant reduction. (**F**) T_2_ relaxation times shows a moderate and significant decrease with Pfirrmann scoring.

**Figure 4:**
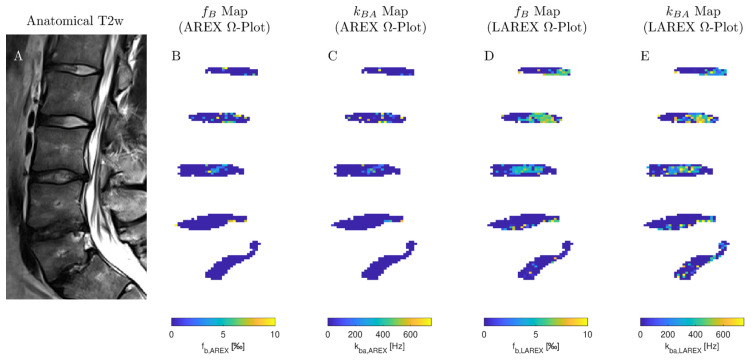
A sagittal image of representative IVDs and the corresponding exchange rate maps in one subject. (**A**) T2-weighted image in the sagittal plane (Pfirrmann scores from head to feet: 2, 2, 3, 4, and 4). (**B**,**C**) Exchange rate maps of the corresponding IVDs using the AREX method. (**D**,**E**) The related exchange rate maps using our proposed LAREX-based Ω-plot method. Figure A6 additionally attaches the T1 and T2 maps as well as a B0 map for the volunteer shown in this figure.

We examined 40 lumbar IVDs from eight volunteers with various stages of IVD degenerations using both the AREX and LAREX approaches. Based on the Pfirrmann classification [30], degeneration in IVDs was quantified as follows: Grade 1 (n = 14), Grade 2 (n = 18), Grade 3 (n = 4), Grade 4 (n = 4), and Grade 5 (n = 0). The mean T_1_ and T_2_ relaxation times were 1.3 ± 0.1 s and 97 ± 16 ms, respectively, which are within the range for our simulations. In the AREX-based Ω analysis, we did not find a significant correlation for neither fractional concentrations (τ = 0.13, *p* = 0.3; Figure 3A) nor exchange rates (τ = −0.26, *p* = 0.04; Figure 3B). It should be mentioned that the fractional concentration with the Pfirrmann scoring increased from f_b,AREX_ = 2.50 ± 1.71‰ (Pfirrmann score 1) to f_B,AREX_ = 2.67 ± 2.02‰ (Pfirrmann score 4) (Table 2). In contrast, we observed a moderate and significant decrease in both f_b_ (τ = −0.49, *p* ≤ 0.0001, Figure 3C) and k_ba_ (τ = −0.48, *p* ≤ 0.0001, Figure 3D) with LAREX-based Ω assessment. Thereby, the fractional concentration decreased from f_b,LAREX_ = 4.96 ± 2.53‰ in healthy IVDs (Pfirrmann score 1) to f_b,LAREX_ = 0.78 ± 0.26‰ in pronounced degenerated IVDs (Pfirrmann score 4) (Table 2). Furthermore, for the in vivo experiments, we observed a significant moderate correlation to disc degeneration for both T_1_ (τ = −0.46, *p* = 0.0002, Figure 3D) and T_2_ (τ = −0.5, *p* ≤ 0.0001, Figure 3D) relaxation times. An anatomical image and the corresponding calculated f_b_ and k_ba_ maps of a representative lumbar spine demonstrate the ability to differentiate between different stages of degeneration using the LAREX-based Ω-plot analysis (Figure 4).

For the AREX-based Ω-plot analyses, an excellent reliability was observed for the determination of fractional concentration with ICC(3,1) = 0.94 (95% CI = 0.90–0.96) and ICC(2,1) = 0.95 (95% CI = 0.91–0.97). In addition, the determination of the exchange rate showed good and excellent agreements, respectively, with ICC(3,1) = 0.87 (95% CI = 0.78–0.92) and ICC(2,1) = 0.95 (95% CI = 0.92–0.97). Similarly, for our proposed LAREX-based Ω-plot analysis, we found good reliability with ICC(3,1) = 0.75 (95% CI = 0.61–0.84) and ICC(2,1) = 0.88 (95% CI = 0.81–0.93) for fractional concentration and ICC(3,1) = 0.85 (95% CI = 0.74–0.91) and ICC(2,1) = 0.78 (95% CI = 0.63–0.87) for exchange rate observation.

## 3. Discussion

This study demonstrates the feasibility of qCEST using a new LAREX-based Ω-plot approach to study fractional concentration in a multi-pool system. We validated our proposed LAREX-based Ω-plot approach for GAG concentration, in silico, in situ (human IVDs from body donors), and in vivo (using IVDs from volunteers). In addition, we have demonstrated the applicability of our method using in silico experiments for APT-qCEST imaging in the brain, amine-qCEST imaging of human blood, and creatine-qCEST analysis in the human brain, considering additional exchanged proton pools in Appendix A. The results indicate that our proposed method substantially improves the accuracy of determining the fractional concentration and the exchange rate.

In contrast to morphological MRI, compositional MRI quantifies tissue composition beyond mere morphology and structure. However, human tissues are complex biochemical systems consisting of numerous components. Thus, while tissue composition can be adequately quantified using compositional MRI, alterations of a specific metabolite within the tissue are far more difficult to assess. Consequently, simple analysis methods like classical MTR_asym_ analysis or quantitative AREX-based Ω-plot analysis reach their limits here. In contrast, our introduced LAREX-based Ω-plot approach overcomes these challenges and allows for the separate analysis of the tissue’s individual functional groups.

We were able to demonstrate that the presence of additional proton pools hampered the classical qCEST approach based on an AREX-based Ω-plot analysis and showed substantial deviations. Our simulations for IVDs showed that AREX substantially and systematically underestimated the fractional concentrations. This occurs in the physiological range from f_B_ = 0.5–5‰ and k_sw_ = 100–500 Hz for the GAG-OH pool, which is clinically relevant. Comparable results were observed by Zhou et al. [31] in a comparison of ideal two-pool phantoms and in situ IVDs. In their study, systematic deviations between in vitro and in situ experiments as a function of pH values were also observed, which were attributed to the non-analyzed pools. In contrast, the LAREX-based evaluation allows for accuracy comparable to that achieved with an ideal system consisting solely of two pools. In addition, we validated the LAREX method by in situ experiments for the quantitative assessment of APT in white and gray matter, the quantitative assessment of amines in human blood, and the assessment of creatine in the brain (Appendix A and Appendix B). Here, we observed comparable results for IVDs. Thus, in future studies, multi-pool methods can be analyzed equivalently using the LAREX approach to two-pool systems. The simulations used in the Appendix A were performed at a field strength of 7T. With the help of the simulations we performed, the LAREX approach we proposed could be successfully validated at 3T (in silico IVD study) and 7T (studies in the Appendix A).

In the in situ experiments, we observed that the AREX-based Ω-plot approach specifically for the determination of fractional concentration did not yield physiological results, and the results analogous to the T_1_ and T_2_ measurements showed no correlation to the histological scoring. Using the LAREX-based Ω-plot approach, we observed a strong and significant correlation (τ = −0.71 *p* = 0.005) of fractional GAG concentration to the histological reference and lowered fractional concentrations compared to the subject collective. Studies of our group demonstrated an age dependence for the MTR_asym_ effect [32]. Since the classical AREX method is based on the MTR_asym_ under different B_1_ field strengths, a decrease in the GAG-induced MTR_asym_ effect such as advanced age leads to an increase in the influence of noise and the other pools. In particular, the NOE#1 effect at −1.8 ppm significantly affects the evaluation of GAG OH protons at one ppm. This may lead to non-physiological results for IVD of advanced age (age of body donors for the in situ measurements: 88.6 ± 8.7 years). The LAREX-based approach allows, due to the Lorentzian correction, not only for the correction of other pools but also a smoothing of the Z-spectrum optimized for CEST experiments [33], which makes this approach suitable for low fractional concentrations. However, it should be considered that Lorentzian analyses have a number of 3 × n (n = number of assumed pools) free parameters. Moreover, CEST exchange problems are nonlinear, which results in the optimization function having not only one minimum but several local minima. If the initial values are chosen unfavorably compared to the input parameters, jumps between minima may occur. However, this is primarily relevant for artificially generated data with only one evaluated pixel. For in situ or in vivo measurements, this effect is compensated by the slight differences between the local pixels and the number of acquired pixels.

In the in vivo experiments performed using the LAREX approach, we observed a moderate and significant decrease in both the fractional concentration (τ = −0.49, *p* ≤ 0.0001) and the exchange rate (τ = −0.48, *p* ≤ 0.0001) of GAGs depending on the IVDs’ degenerative stage. The AREX-based evaluation, on the other hand, showed no significant correlations and determined a non-physiologically increase in GAG concentrations with progressive degeneration. With LAREX, a fractional concentration of 4.96 ± 2.54‰ was observed in IVDs without any degeneration (Pfirrmann grade 1) and 0.78 ± 0.26‰ in IVDs with advanced degeneration (Pfirrmann grade 4). Considering three exchanging OH protons at one GAG [10] and a water concentration of about 80% in IVDs [34], the corresponding concentrations are 150 mM (Pfirrmann grade 1) and 25 mM of GAG concentration (Pfirrmann grade 4). Iatridis et al. determined GAG concentrations of 250 µg ± 134 GAG/mg dry IVD tissue [34], which relates to a 50–150 mM GAG concentration, which is consistent with our results. Moreover, in our in vivo experiments, we observed about two times higher fractional concentrations than in the in situ experiments, which agrees with previous studies [10,35], which showed a decrease in GAG as a function of age and associated progression of IVD degeneration. Furthermore, in previous studies, 66–100 mM GAG concentrations were found in articular cartilage [10,35]. Therefore, the cutoff and baseline values of the Lorentzian analysis we presented could be adopted to examine articular cartilage for alterations in GAG concentration in future studies. Moreover, regional differences in healthy IVDs were observed in our in vivo experiments (Pfirrmann ≤ 3, Figure 4), which might be due to the differences between nucleus pulposus and annulus fibrosus. Previous studies have also observed these regional differences using MTR_asym_ [3,4,32].

Numerous previous studies have demonstrated the potential for deep learning in medical imaging and post-processing [36,37,38]. Among others, Zaiss et al. showed the potential for deep learning in qCEST imaging [39]. Recently, Huang et al. published two fast and accurate ways to generate CEST/AREX contrast maps using DeepCEST and DeepAREX in animal experiments [40]. Based on those studies and our results, neural networks could be developed to eliminate the influence of other pools. Thus, our results suggest a promising new approach to qCEST imaging. Further longitudinal studies could periodically examine subjects or animal models to determine if the early deterioration can be detected.

However, our study has some limitations. First, the long measurement time of 84:35 min (in situ) and 36:15 min (in vivo) for a one-slice sequence must be mentioned. This is foremost because regular CEST experiments require long TR times, thus guaranteeing T_1_ relaxation time decay. In addition, qCEST analysis requires an RF saturation time (10 s in our study) to reach a steady-state and at least three different CEST experiments with different B_1_ amplitudes to perform the Ω-plots. By using dual-gradient echo strategies [41], simulated multi-slice gradient echo (GRE) sequences [42], phase-shifted multiplanar CEST—fast imaging with steady-state free precession (FISP) sequences [43], ultrafast one-shot acquisition [44], compressed sensing [45], and other imaging techniques, this limitation could be addressed in future studies [46]. Second, the two IVD MR studies we performed (in situ and in vivo) have only limited comparability. As these experiments were conducted at different temperatures (20 °C in situ and 37 °C in vivo), there may be systematic differences between the experiments due to temperature differences, which might affect the accuracy of LAREX and AREX. However, it should be noted that temperature changes do not change the linear dependence of the CEST effect on concentration [13]. Therefore, the increased accuracy of the LAREX approach compared to the AREX approach would not change by adjusting the temperature. Third, the present method relies on the fact that Lorentz functions can describe the various CEST effects. Previous studies have shown that Lorentz functions cannot represent rapidly exchanging metabolites [47]. Therefore, the accuracy of the LAREX method depends on the exchange rate of the metabolites of the multi-pool system, and as a result, LAREX is not necessarily better than AREX for all CEST metabolites [47]. Fourth, the application of the Lorentz fitting not only enables a reduction of the spurious effects of other pools but also has a CEST adaptive denoising effect. Contrary to this, the AREX-based application only used the NLM denoising filter [48], which is not optimized for CEST. Fifth, our in situ assessments were only semi-quantitative concerning GAG concentrations. Recently, Kubaski et al. demonstrated a rapid, sensitive, and accurate measurement of GAG and all isomers by mass spectrometric detection analysis [49]. Thus, in the subsequent studies, the sensitivity of the LAREX approach could be investigated in more detail than the laborious spectroscopic methods. Sixth, the in situ and in vivo studies we performed were performed at only a single time point. In further studies, detailed reproducibility measurements and longitudinal studies over a long period of time are needed to detect degenerative processes at multiple time points and to investigate the degenerative changes. Seventh, in the in situ and in vivo studies we performed using LAREX, we observed a moderate change in k_ba_ as a function of the Pfirrmann grade. Previous studies have shown that pH changes in the IVD due to degeneration [31,50]. Subsequent in situ or in vitro studies will need to infer changes of k_ba_ as function of pH-values. Eighth, the application of the LAREX approach at 7T and its use outside IVDs were validated in our study only by in silico experiments. Further in situ and in vivo studies, guided by the IVD studies we performed, are needed to validate the promising LAREX approach further and prepare it for clinical applicability.

## 4. Materials and Methods

### 4.1. Study Design

This prospective feasibility study included sequential in silico, in situ, and in vivo CEST MRI assessments and was, thus, conducted in three consecutive steps: (1) implementation and validation of the novel LAREX approach for quantitative multipool CEST-MRI evaluation based on a Lorentzian adaptation and AREX-based Ω-plot analyses using in silico studies (2) evaluation of the newly implemented LAREX approach using in situ experiments and histological referencing of GAG content in human IVDs, and (3) demonstration of the in vivo applicability in eight subjects with different stages of DDD.

Written informed consent was obtained from all body donors as well as all volunteers. The study was approved by the local ethics committee (Ethics Committee of the Medical Faculty of Heinrich Heine University, Düsseldorf, Germany, study numbers: in situ 2021-1528 and in vivo 2019-551).

### 4.2. MR Imaging

All MRI measurements were performed on a clinical 3T MRI scanner (MAGNETOM Prisma, Siemens Healthineers, Erlangen, Germany) with a dedicated 15-channel knee coil (Tx/Rx Knee 15 Flare Coil, Siemens Healthineers) for the in situ experiments or with the integrated 24-channel spine coil (Spine Matrix coil, Siemens Healthineers) for the in vivo experiments.

For morphologic reference imaging, a sagittal T1-weighted (T1w) turbo spin-echo (TSE) sequence for the alignment of the compositional sequences as well as a sagittal T2-weighted (T2w) TSE sequence were used. For bio-sensitive MRI, T_1_ and T_2_ mapping sequences, as well as CEST sequences, were obtained; for T_1_ mapping, an inversion recovery TSE sequence with seven inversion times (TIs: 25–3000 ms) (Table 3), while for T_2_ mapping, a spin-echo (SE) sequence with 20 different echo times was obtained (TEs: 9.7–194 ms) (Table 3). Furthermore, seven (in situ) or three (in vivo) CEST sequences with different high-frequency pulse amplitudes (B_1_) were acquired (Table 3). The saturation module of our CEST sequence used Gaussian RF pulses. To this end, a total of 64 saturated images were acquired at different saturation frequencies around the water resonance between −5 and 5 ppm, with a reference image at 300 ppm, and we systematically increased the B_1_ pulse amplitude from 0.6 to 1.2 µT in 0.1 µT (in situ) or 0.3 µT (in vivo) steps. In addition, the pulse duration (t_p_ = 100 ms), pulse delay (t_d_ = 100 ms), and number of presaturation pulses (n_p_ = 40) were chosen to achieve a steady-state condition. For the B_0_ inhomogeneity correction [51], a Water Saturation Shift Referencing (WASSR) sequence was acquired with t_p_ = 25 ms, t_d_ = 25 ms, n_p_ = 1, B_1_ = 0.2 µT, 22 dynamics, and frequency offsets between −1 and 1 ppm [52]; otherwise, the same sequence parameters were used for the CEST sequence.

### 4.3. In Silico Study

In accordance with previous studies, we simulated the Z-spectra based on the Bloch–McConnell equations using a house intern modified version of the open-source MATLAB (MatlabR2020b, Natick, MA, USA) script published by Zaiss et al. (Link to download: https://github.com/cest-sources/BM_sim_fit/, accessed on 16 March 2021) [13,19,53,54,55]. We evaluated the accuracy of the AREX- and LAREX-based Ω-plot analyses as a function of fractional GAG concentration f_b_ and hydroxyl proton exchange rate k_ba_. Previously, Stabinska et al. showed that AREX-based Ω-plot analyses are varyingly accurate as a function of f_b_ and k_ba_ [17]. Therefore, we performed additional ideal simulations for a two-pool system consisting solely of water and the substrate to compare our multi-pool results. The same MR (TR = 2500 ms, TE = 3.5 ms) and presaturation parameters (n_p_ = 40, t_p_ = 100 ms, t_d_ = 100 ms, presaturation pulse shape = Gaussian, Δω = 5 ppm) as well as a field strength of 3T were used for the simulations and the subsequent in situ and in vivo reference studies. GAG concentrations were systematically varied from 10 mM to 250 mM (f_b_ = 0.23–8.52‰) in an extended physiological range [19,31,56]. Accordingly, OH proton exchange rates k_ba_ were varied between 50 and 1000 Hz as described in previous studies [31,57]. A water concentration of 80% in IVDs was assumed, as shown by Baldoni et al. [29]; the other parameters used are listed with references in Table 1.

We also performed further in silico analyses, which can be found in Appendix A. In these analyses, we investigated our proposed LAREX-based Ω-plot approach for APT qCEST imaging in white matter (Appendix A Figure A1) and gray matter (Appendix A Figure A2), APT qCEST imaging of blood (Appendix A Figure A3), and creatine qCEST imaging in the human brain (Appendix A Figure A4) to illustrate its clinical applicability to a wide range of clinically relevant multi-pool systems.

### 4.4. In Situ Study

**Human lumbar intervertebral disc cadavers:** The local Institute of Anatomy I (Heinrich Heine University, Düsseldorf, Germany) provided eleven freshly frozen human IVDs from three body donors for the in situ measurements. At the time of death, the mean age of the body donors was 88.6 ± 8.7 years (range 82–101 years), one was female, and two were male. Before each MRI examination, specimens were thawed and warmed to room temperature for at least 24 hours.

**In situ MR imaging:** All specimens were positioned centrally in the dedicated knee coil. In addition, mechanical positioning devices such as sandbags were used to fix the specimens in the coil. A baseline morphological T1w sequence was acquired to align the bio-sensitive imaging sequences. Subsequently, the specimens were examined with seven different B_1_ field strengths as described above. Consistent with previous studies, a WASSR sequence was used for B_0_ inhomogeneity correction [3,8,19].

**Histological preparation:** Following the MRI examination, the specimens were subjected to a standard histological procedure [58]. For this purpose, the IVDs and adjacent vertebrae were decalcified and fixed in Ossa fixona (Diagonal, Münster, Germany), dehydrated, and embedded in paraffin. IVDs were then cut along the mid-sagittal plane. The initial condition of the discs was semi-quantitatively scored according to Thompson et al. [29]. Thompson scoring. as the gold standard, allows for a full assessment of the degenerative stage of discs (score 1—no degeneration, score 5—fully degenerative IVD). T.J.F., who has 33 years of experience in musculoskeletal histopathology, assessed each tissue sample individually. In addition, IVDs were cut into 5-μm-thick slices and stained with Safranin O to validate the Thompson assessments [59]. A conventional light microscope (Motic Easy Scan Infinity 100, MoticEurope, Barcelona, Spain) and dedicated software (Motic^®^ Images Devices MoticEurope, Barcelona, Spain) were used for validation; deviating staining compared to the Thompson score would lead to IVD exclusion.

### 4.5. In Vivo Study

The in vivo study was carried out on eight volunteers (mean age: 29 ± 4 years; age range: 24 to 35 years; six male, two female) with various stages of disc degenerations. All subjects were positioned headfirst and supine in the center of the MRI scanner. Morphological sequences (i.e., T1w- and T2w-sequences) were obtained for clinical assessment, such as Pfirrmann scoring of IVDs [30]. To determine the Pfirrmann degree of IVD degeneration, signal intensity on T2-weighted MR images was used to estimate water content with morphologic parameters on a scale of 1 (no degeneration) to 5 (completely degenerated). To reduce scanning time, only three of the CEST sequences used for the in vivo experiments (B_1_ = 0.6, 0.9, and 1.2 µT, t_p_ = 100 ms, n_p_ = 40, pulse shape = Gauss) were acquired. For the B_0_ inhomogeneity correction, a WASSR sequence was performed according to the in situ measurements as previously described.

### 4.6. LAREX: Lorentzian-Corrected Apparent Exchange-Dependent Relaxation

**Figure 5:**
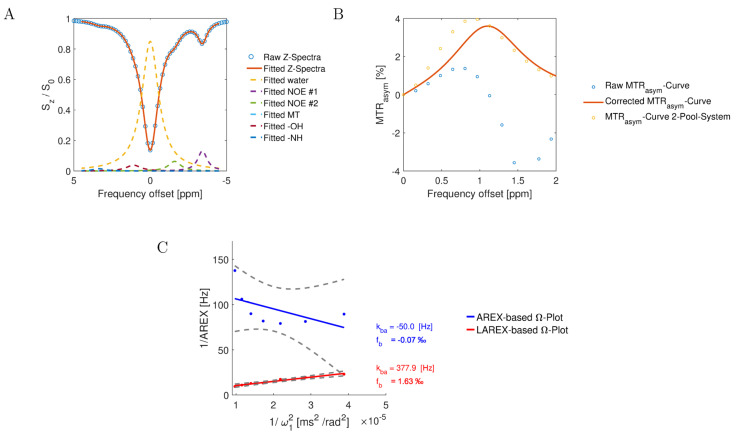
Graphical representation of an Ω-plot analysis using AREX and LAREX data, with Bloch–McConnell simulated Z-spectra for a 6-pool IVD exchange system. For the analysis, the parameters of the hydroxyl pool were f_b_ = 1.8‰ and k_ba_ = 400 Hz. The other pools’ parameters are outlined in Table 3. (**A**) Plot of an exemplary Z-spectra (blue circles) at B1 = 0.7 µT and the fitted Z-spectra (red line), and the Lorentz fits of each pool (colored dashed lines). (**B**) The plot of the MTR_asym_ curve for the simulated raw Z-spectra (blue circles, Raw MTR_asym_-Curve), the MTR_asym_-Curves based on the Lorentz corrected Z-spectra to a two-pool system of water and Gag-OH (red line, Corrected MTR_asym_-Curve), and the ground truth MTR_asym_-Curve with based on a Bloch–McConnell simulation with only 2-pools (yellow circles, _MTRasym_-Curve 2-Pool-System). (**C**) Visualization of the AREX (blue line) or LAREX-based (red line) Ω-plots. The gray dashed lines illustrate the 95% confidence interval of the linear omega plots.

The Lorentzian-corrected apparent exchange-dependent relaxation Ω-analysis is an extension of the classical AREX approach for systems with multiple pools, using Lorentzian analyses to eliminate the influence of other proton pools (Figure 5). Analogous to the AREX approach, Z-spectra were first normalized. Subsequently, the different overlapping proton pools affecting the CEST effect are fitted and corrected by Lorentzian analyses (Figure 5). Analogous to the AREX approach, Z-spectra were first normalized (step 1). Next, the different overlapping proton pools affecting the CEST effect are fitted by Lorentzian analyses (step 2, Figure 5A). Subsequently, 2-pool Z-spectra were calculated, which are composed of a superposition of the calculated water pool and the investigated metabolite pool, and MTR_asym_ curves were calculated (step 3, Figure 5B). Finally, the Z-spectra were evaluated analogously to the classical AREX approach, where the measured Z-spectra were replaced by the corrected Z-spectra (step 4, Figure 5C). Therefore, for the IVDs experiments, the Z spectra were fitted considering the nuclear Overhauser enhancement—NOE #1 (−1.6 ppm), NOE #2 (−3.5 ppm), MT, spillover, OH, and NH effects. Due to the large number of free hyperparameters that arise in a multi-pool system, we used a user-defined error function (Equation (1)). For curve fitting, we used the MATLAB optimization solver fmincon (find minimum of constrained nonlinear multivariable function) with a state-of-the-art sequential quadratic (SQP) algorithm. [60]. Following this correction mechanism, the Z-spectrum corresponds only to a two-pool system, which can be evaluated analogously to previous studies using AREX [17,22].

**Figure A5:**
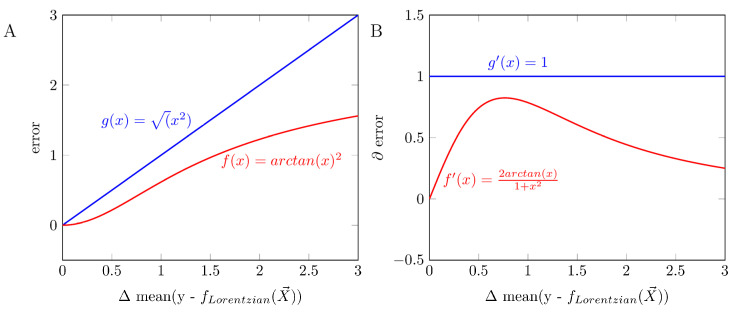
Comparison of arctan as an error function versus the commonly used Root Mean Squared Error (RMSE) function. (**A**) Calculated error as a function of the mean deviation. (**B**) The course of the gradient of the error function as a function of the mean deviation. Here, the arctan shows an adaptive adjustment of the error gradient, which is especially advantageous for optimizing many hyperparameters.

The error function we proposed is based on the arctangent, which had proven itself as an error function under the minimization of numerous hyperparameters in machine learning [61].

(1) $$error\left(\vec{X}\right)={\left(arctan\left(mean\left(y-f_{Lorentzian}\left(\vec{X}\right)\right)\right)\right)}^{2}$$

where *y* corresponds to the simulated or measured values of the Z-spectra, *f_Lorentzian_* corresponds to the Lorentzian function, and $\vec{X}$ corresponds to the hyperparameters to be optimized. Compared to the commonly used Root-Mean-Squared-Error, the error function differs by an adaptive gradient around zero (Appendix B Figure A5). Since the Z-spectra are normalized to y-values between 0 and 1, just the range smaller than 1 is decisive for the behavior of the error functions. Table 4 shows the fitting parameters we used. For the Lorentzian analysis of the Z spectrum, the different pools are not fitted to a fixed frequency offset. Instead, they are determined in a frequency range (Table 4); these deviations from the presumed frequency offset were corrected where necessary by replacing the frequency in the Lorentzian function with the assumed frequency. Consequently, no deviations from the assumed and evaluated frequency offsets are present in the corrected Z spectra of the LAREX evaluation. The subsequent calculations of the MTR_Rex_ maps, AREX maps, and Ω-plots were consistently performed.

### 4.7. MR Image Analysis

For further data analysis, the acquired in situ and in vivo MR data were segmented independently by two experienced radiologists, L.M.W. (5 years of musculoskeletal imaging experience) and D.B.A. (6 years of musculoskeletal imaging experience), using ITK Snap software (v3.8.0, Cognitica, Philadelphia, PA, USA) [62]. L.M.W. segmented the ROIs twice (i.e., six weeks apart) to assess the intra-reader reliability of the techniques studied, whereas D.B.A. segmented the data only once to determine inter-reader reliability. In addition, D.B.A. assessed the Pfirrmann grade for the in vivo measurements [30]. Additionally, tissue properties were further analyzed using in-house developed MATLAB scripts. T_1_ maps were created by fitting the IR measurement data with the non-linear least-squares method as a function of inversion delay (TI): S(TI)~S_0_ (1 − 2 × exp(−TI/T1)), where S_0_ is the equilibrium signal. T_2_ maps were consistently calculated pixel-wise as a function of echo time (TE): S(TE)~S_0_ × exp(−TE/T2). Based on the WASSR measurements, the pixel-wise B_1_ inhomogeneity offset maps were calculated. The CEST data were normalized to the signal of the first acquired frame with a frequency offset of 300 ppm. Using a custom MATLAB script validated in previous in silico and in vitro studies, the subsequent Ω-plots were performed [17]. To this end, the MR images were first denoised using the non-local means (NLM) filter [48], and Z-spectra were frequency corrected using the offset maps; the MTR_Rex_ maps with inverse asymmetry; and the AREX maps determined with the frequency shift of 1 ppm specific to hydroxyl protons. Subsequently, the relaxation-compensated Ω-plot analysis was calculated. In addition, an R_2b_ of 100 Hz was assumed for the hydroxyl protons bounded on GAG in accordance with Singh et al. [28].

### 4.8. Statistical Analysis

Statistical analyses were made by K.L.R. in R (v4.1.3, R Foundation for Statistical Computing). The Kendall-Tau rank correlation coefficient (τ) was determined to investigate possible statistical relationships between the determined surrogate parameters f_b_ and k_ba_ as a function of the histological Thompson classification (in situ) and Pfirrmann scoring (in vivo). The tau effect size was classified as low (0.1–0.3), medium (0.3–0.5), and strong (>0.5), according to Cohen et al. [63]. To measure relative reliability, the intraclass correlation coefficient (ICC) was used with a 95% confidence interval and was classified according to Koo et al. as poor (ICC < 0.5), moderate (0.5 ≤ ICC < 0.75), good (0.75 ≤ ICC < 0.9), and excellent (ICC ≥ 0.9) [64]. For determining inter-rater reliability, the ICC(2,1) was used, and intra-rater reliability was determined using the ICC(3,1) [65].

Due to the experimental design of this study, the significance level was set from *p* ≤ 0.05 to an adjusted *p* ≤ 0.00625 according to the conservative alpha adjustment method Bonferroni [66]. This “low” significance level prevented alpha error inflation while simultaneously maintaining statistical power.

## 5. Conclusions

In our study of qCEST imaging in multi-pool models, we validated our proposed LAREX method using in silico experiments for a wide range of medically relevant systems such as human IVDs, the white and gray matter of the visual cortex, and human ex-vivo blood. Moreover, we have therefore shown that Lorentz analyses can be used to extract 2-pool spectra from multi-pool spectra. These calculated 2-pool spectra yield results comparable to real 2-pool spectra in an omega plot analysis. Furthermore, we could transfer the results to in situ and in vivo studies. Therefore, for the first time, it is possible to quantitatively investigate multi-pool systems by means of fractional concentration and exchange rate and by using qCEST with the LAREX approach.

## Figures and Tables

**Figure A7:**
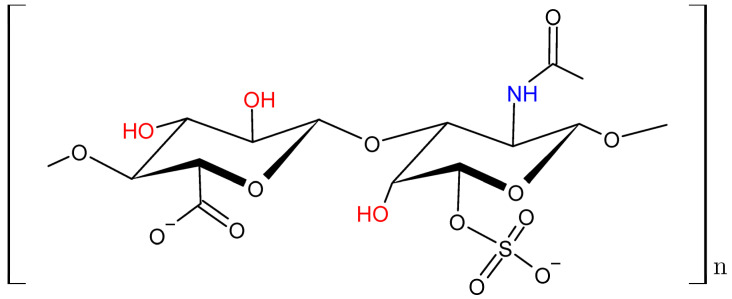
The molecular formula of chondroitin sulfate, the most abundant glycosaminoglycan in cartilage. The three exchanging hydroxyl groups (-OH) are highlighted in red, and the one exchanging amide group (-NH) in blue.

**Table 1:** qCEST parameters with references for the six-pool IVD numerical simulation with solute concentration (f), the solute–water exchange rate (k_ba_), longitudinal relaxation time (T_1_), transversal relaxation time (T_2_), and solute resonance frequency offset (Δ). Figure A7 also shows chondroitin sulfate as structural formula, showing the 3:1 ratio of hydroxyl to amide protons.

	Water [8]	Hydroxyl [28]	Amide [25]	NOE #1 [25]	NOE #2 [25]	MT [25]
Pool	A	B	C	D	E	F
T_1_ (ms)	1306	T_1,a_	T_1,a_	T_1,a_	T_1,a_	T_1,a_
T_2_ (ms)	134	10	2	1	0.5	0.015
f	1	variable	1/3 × f_B_	0.003	0.007	0.1
k_ba_ (Hz)	-	variable	50	50	50	25
Δ (ppm)	0	1	3.5	−1.6	−3.5	−2.3

Abbreviations: NOE—nuclear Overhauser enhancement; MT—magnetization transfer, T_1,a_—longitudinal relaxation time of the pool A, f_B_—fractional concentration of Pool B.

**Table 2:** Evaluation of the imaging characteristics of fractional concentration (f_b_) and exchange rate (k_ba_) for the degenerative stage (Pfirrmann score). Each value is expressed as mean and standard deviation.

Parameter	Pfirrmann Score	AREX-Based Ω-Analyses	LAREX-Based Ω-Analyses
f_b_ [‰]	1	2.49 ± 1.71	4.96 ± 2.54
	2	2.48 ± 1.68	3.04 ± 1.52
	3	2.48 ± 1.67	1.85 ± 1.43
	4	2.67 ± 0.38	0.79 ± 0.26
k_ba_ [Hz]	1	215.7 ± 118.2	292.2 ± 125.5
	2	175.2 ± 127.1	199.5 ± 76.0
	3	152.6 ± 78.7	166.7 ± 57.1
	4	80.9 ± 76.5	122.6 ± 36.4

**Table 3:** Magnetic resonance imaging sequence parameters.

	T1w TSE	T2w TSE *	T1 Mapping ^A^	T2-SE Mapping	CEST In Situ ^C^	CEST In Vivo ^D^	WASSR
Orientation	sag	sag	sag	sag	sag	sag	sag
TE (ms)	9.8	95	10	^B^	3.5	3.5	3.5
TR (ms)	650	3500	6000	1000	2500	2500	2500
Slices	15	15	1	1	1	1	1
Slice Thickness (mm)	3	4	4	4	4	6	4/6
FOV (mm × mm)	300 × 300	260 × 260	200 × 200	200 × 200	200 × 200	200 × 200	200 × 200
Image matrix (pixel)	384 × 384	384 × 384	128 × 128	128 × 128	128 × 128	128 × 128	128 × 128
Flip angle (°)	150	160	180	180	15	15	15
Turbo Factor	109	17	11	na	na	na	na
GRAPPA	2	na	2	na	na	na	na
Duration (min:s)	1:12	3:46	8:38	1:12	84:35	36:15	3:43
*—only in vivo ^A^—TI = 25, 50, 100, 500, 1000, 2000 and 3000 ms ^B^—TE = 9.7 ms to 197 ms with a step size of 9.7 ms ^C^—B_1_ = 0.6, 0.7, 0.8, 0.9, 1.0, 1.1, 1.2 µT; t_p_ = 100; t_d_ = 100 ms and n_p_ = 40 ^D^—B_1_ = 0.6, 0.9, 1.2 µT; t_p_ = 100 ms; t_d_ = 100 ms and n_p_ = 40							

Abbreviations: T1w—T1 weighted; TSE—turbo spin echo; SE—spin echo; CEST—chemical exchange saturation transfer; WASSR—water saturation shift referencing; sag—sagittal; TE—echo time, TR—repetition time, FOV—field of view; na—not available; GRAPPA—Generalized Autocalibrating Partial Parallel Acquisition; TI—inversion time; B_1_—high-frequency field strength; t_p_—pulse duration; t_d_—interpulse delay; n_p_—number of saturation pulses.

**Table 4:** Starting points and boundaries of the amplitude (A), width (W), and offset (Δ) of the six-pool Lorentzian fit, which we used for our in silico, in situ, and in vivo studies of IVDs. Furthermore, we used a custom loss function (Equation (1)).

	A_water_	W_water_	Δ_water_	A_amide_	W_amide_	Δ_amide_	A_hydroxyl_	W_hydroxyl_	Δ_hydroxyl_
Start	0.85	2	0	0.01	1	3.5	0.01	1	1
Lower	0.5	1	−0.5	0	0.2	3	0	0.5	0.6
Upper	1.1	6	0.5	0.1	3	4	0.1	2	1.4
	A_NOE #1_	W_NOE #1_	Δ_NOE #1_	A_NOE #2_	W_NOE #2_	Δ_NOE #2_	A_MT_	W_MT_	Δ_MT_
Start	0.001	1	−1.6	0.03	1	−3.5	0.1	10	−2.3
Lower	0	0.5	−2.5	0	0.5	−5	0	8	−3
Upper	0.1	3.5	−0.5	0.2	4	−3	0.5	20	−2

**Figure A6:**
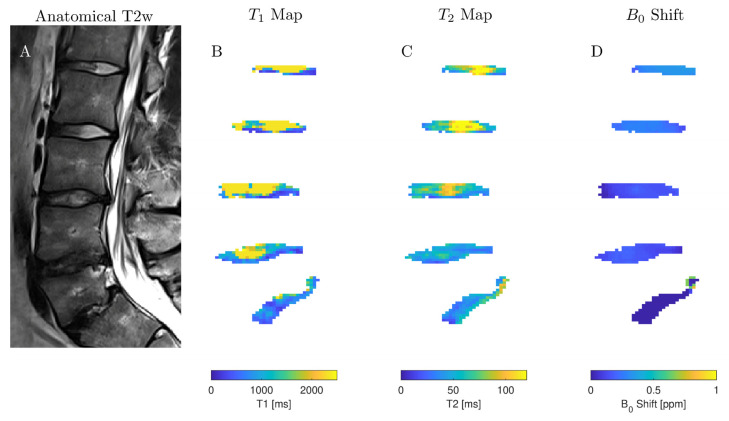
A sagittal T2-weighted (T2w) image (**A**) of the volunteer shown in Figure 4 and the corresponding longitudinal relaxation time (T1) map (**B**), transverse relaxation time (T2) map (**C**), and B0 field displacement map (**D**).

## Data Availability

Data and evaluation scripts can be provided by the authors upon reasonable request.

## Appendix A

The following figures demonstrate the results for the transferability of our presented LAREX-based Ω-plot approach for other medically relevant multipool systems outside of human IVDs. The simulation parameters used for the longitudinal relaxation time (T_1_), transverse relaxation time (T_2_), fractional substrate concentration (f), and substrate exchange rate with the water pool (k_ba_) are given for each pool in the corresponding figure captions below.
